# Reviewed article: Silva MAL, Mendes LL, Leite MA, Rocha LL, Borges CA, Levy RB, Louzada MLC. Food purchasing places classification system based on the Dietary Guidelines for the Brazilian Population: Locais-Nova. Epidemiol Serv Saude. 2025:34;20240361.

**DOI:** 10.1590/S2237-96222025v34e20240361.a

**Published:** 2025-04-11

**Authors:** Patrícia Freitas

**Affiliations:** Universidade Federal dos Vales do Jequitinhonha e Mucuri, Departamento de Nutrição proxy

**Table:** 

Rating	Excellent	Good	Average	Below average	Poor
Interest	✓				
Quality	✓				
Originality	✓				
Overall	✓				

**Table:** 

Questionnaire	Yes	No	Not applicable
Does the manuscript contain new and significant information to justify publication?	✓		
Does the Abstract (Summary) clearly and accurately describe the content of the article?	✓		
Is the problem significant and concisely stated?	✓		
Are the methods described comprehensively?	✓		
Are the interpretations and conclusions justified by the results?	✓		
Is adequate reference made to other work in the field?	✓		

## Comments

O artigo é bem escrito, aborda um tema relevante para a área e apresenta resultados inovadores que certamente contribuirão para o monitoramento dos ambientes alimentares e para o desenvolvimento de políticas públicas. A seguir, são apresentadas sugestões de alterações pontuais e discussões importantes.

**Introdução**

No penúltimo parágrafo é apontada uma limitação na avaliação de ambientes alimentares feita pela Câmara Interministerial de Segurança Alimentar e Nutricional em 2018. Essa limitação poderia ser melhor explicada. Ficou vago e não está claro para o interlocutor. Na discussão o assunto é retomado, mas lá pode ser dada ênfase no avanço que a metodologia proposta representa.

**Métodos**

A análise estatística precisa ser detalhada. Quais os testes utilizados? Quais comparações feitas?

**Resultados**

Por exemplo, os resultados apresentados no segundo parágrafo foram significativos?

O termo “fontes exclusivas” utilizado ao longo do texto pode causar confusão no interlocutor. Parece que os locais vendem apenas esse tipo de alimento. Deve ser avaliada a substituição do termo para outro a fim de evitar confusão.

**Discussão**

A estratificação por regional foi muito relevante para identificar as diferenças entre os contextos. Mas outras características como o porte do município e localização do domicílio urbana ou rural também poderiam impactar nos resultados. Seria importante os autores incluírem uma breve discussão a respeito.

Na página 7 os autores discutem sobre a propaganda de alimentos dentro dos estabelecimentos. As referências apresentadas limitam-se a análise de folhetos. Mas outras estratégias de publicidade observada dentro de estabelecimentos comerciais vão ser relevantes para as escolhas alimentares. Seria interessante trazer dados sobre essa abordagem. Inclusive como são utilizadas distintamente entre ultraprocessados e in natura.

Os autores afirma que o indicador de “locais de aquisição fonte exclusivamente de alimentos que devem ser a base da alimentação” identifica locais que não possuem concorrência entre alimentos que devem ser a base da alimentação e os que devem ser evitados. Será que podemos afirmar que não há concorrência apenas pela classificação de “fonte”? Isso não significa que não há AUP lá. Além disso, existem as características do ambiente do consumidor que podem impactar.

